# Auditory Neuropathy after Damage to Cochlear Spiral Ganglion Neurons in Mice Resulting from Conditional Expression of Diphtheria Toxin Receptors

**DOI:** 10.1038/s41598-017-06600-6

**Published:** 2017-07-25

**Authors:** Haolai Pan, Qiang Song, Yanyan Huang, Jiping Wang, Renjie Chai, Shankai Yin, Jian Wang

**Affiliations:** 1 Department of Otolaryngology, Affiliated Sixth People’s Hospital, Shanghai Jiao Tong University, 600 Yishan Road, Shanghai, China 200233; 2 Key Laboratory for Developmental Genes and Human Disease, Ministry of Education, Institute of Life Sciences, Southeast University, Nanjing, 210096 China; 3 School of Human Communication Disorders, Dalhousie University, 1256 Barrington St. Dalhousie University, Halifax, NS B3J1Y6 Canada

## Abstract

Auditory neuropathy (AN) is a hearing disorder characterized by normal cochlear amplification to sound but poor temporal processing and auditory perception in noisy backgrounds. These deficits likely result from impairments in auditory neural synchrony; such dyssynchrony of the neural responses has been linked to demyelination of auditory nerve fibers. However, no appropriate animal models are currently available that mimic this pathology. In this study, Cre-inducible diphtheria toxin receptor (*iDTR* ^*+/+*^) mice were cross-mated with mice containing Cre (*Bhlhb5-Cre* ^*+/−*^) specific to spiral ganglion neurons (SGNs). In double-positive offspring mice, the injection of diphtheria toxin (DT) led to a 30–40% rate of death for SGNs, but no hair cell damage. Demyelination types of pathologies were observed around the surviving SGNs and their fibers, many of which were distorted in shape. Correspondingly, a significant reduction in response synchrony to amplitude modulation was observed in this group of animals compared to the controls, which had a Cre^−^ genotype. Taken together, our results suggest that SGN damage following the injection of DT in mice with *Bhlhb5-Cre* ^*+/−*^ and *iDTR* ^*+/−*^ is likely to be a good AN model of demyelination.

## Introduction

Auditory neuropathy (AN) is a hearing disorder characterized as having normal cochlear microphonic (CM) potentials and otoacoustic emissions (OAEs), but largely reduced or missing auditory brainstem responses (ABRs). Behaviorally, subjects with AN typically show deficits in temporal processing and signal detection in noise that are disproportional to the amount of threshold shifts^1–4^. These findings suggest that AN is not resulted from the pathologies of OHCs. While the mechanism is not entirely clear, the pathological sites of AN may include inner hair cells (IHCs) (termed as presynaptic AN)^5^, the synapses between IHCs and spiral ganglion neurons (SGNs)^6, 7^ and the SGNs (termed as postsynaptic AN)^8, 9^. Since the major functional disorder in human subjects with AN is a lack of synchrony in signal processing^10^, demyelination of auditory nerve fibers (ANFs) is predicted to be a major pathology in AN. However, no such damage has been identified as the core pathology for AN in human subjects except where AN is part of hereditary disorders^8^.

The present understanding of neural dyssynchrony in AN and its relationship with demyelination is limited by the lack of appropriate animal models. Several studies have attempted to develop such models. One study used a jaundiced Gunn rat model, in which neuropathy-like symptoms were induced by a high concentration of bilirubin due to the lack of glucuronosyl transferase in rats^11^. Hyperbilirubinemia created at the neonatal age in this model appeared to reduce the size of SGNs with a lack of myelinated nerve fibers. Since the pathologies were examined only at 18 days after birth, it remains unclear whether the damage is permanent. In addition, no data were provided on the functional consequences of this pathology to justify it as an AN model. In another work, selective damage to IHCs and consequent degeneration of SGNs were observed in chinchillas using carboplatin. This unique pathology was initially reported in the early 1990s; it was hoped that it would produce a model for AN^12^. However, detailed observation failed to show a loss of ANF synchrony in such animals^13^. Gene engineering models have been introduced for selective damage of SGNs. For example, demyelination of SGNs and their nerve fibers occurs in mice with consititutive saposin B knock-out^9^. Application of glutamate or its agonists (such as amino hydroxy methyl isoxzzale propionic acid [AMPA])^14, 15^, as well as ouabain^16^, has also been reported to result in selectiveSGN death at an appropriate dose. A very recent study in which 95% of SGNs were killed using ouabin found a signficant increase in central gain that fully compensated for the auditory response amplitude at a cortical level^17^. Unfortunately, no evidence of demyelination or dyssynchrony in the auditory response was reported in any of these models.

To generate a dyssynchrony-type AN model, we cross-mated mutant mice that had the Cre-inducible diphtheria toxin (DT) receptor (*iDTR* ^*+/+*^) with mice that had a SGN selective Cre (*Bhlhb5-Cre* ^*+/−*^). SGNs in offspring mice of the first generation that are double positive (*Cre* ^*+/−*^, *iDTR* ^*+/−*^) are sensitive to DT^18^; therefore, it is an inducible model for selective lesions to SGNs. We examined the morphology and cochlear functions to verify whether this is an appropriate model for characterizing dyssynchrony in human AN.

## Results

### Auditory function

**Figure 1:**
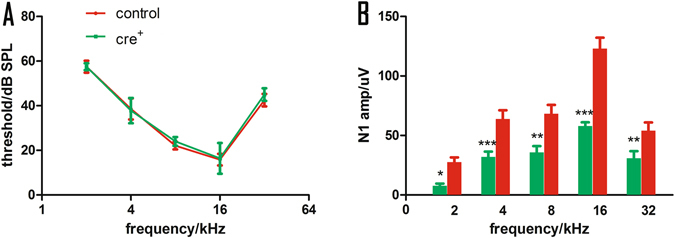
The impact of DT-induced damage on ABR and CAP. (**A**) ABR threshold-frequency curves. (**B**) CAP amplitudes measured in responses to tone bursts of different frequencies at 90 dB SPL. There is no significant between-group difference in ABR threshold. However, the CAP amplitude is much smaller in Cre^+^ group. *p < 0.05,**p < 0.01, and ***p < 0.001.

Since a DT injection induces SGN lesions only in mice with Cre, Cre^−^ mice were used as control animals. The impact of SGN death induced by DT application on auditory function was evaluated in terms of the ABR threshold, compound action potential (CAP) input/output functions in response to tone-bursts, and CAP responses to the amplitude modulation (AM) signals. Because the loss of SGNs reached a plateau at 7 days post injection, the auditory evaluations were performed only at this time point. Figure 1A shows that the ABR thresholds largely overlapped between the two groups with and without Cre. The frequency-averaged thresholds were 39.71 ± 2.28 and 39.86 ± 1.83 dB SPL in the control and *Cre* ^*+*^ groups, respectively, and no significant differences were evident between the groups, as shown by a grouped t-test (t = −0.05, *p* = 0.9618; n = 7 in each group). Figure 1B shows the CAP amplitude measured at 90 dB SPL across frequencies demonstrating an approximately 43–53% reduction in the *Cre* ^*+*^ group compared to the control group. For example, the amplitudes at 16 kHz were 57.94 ± 3.16 μV and 123.08 ± 9.1 μV in the *Cre* ^+^ and control groups, respectively. A two-way ANOVA performed against the factor of group using the factor of frequency as a covariant revealed a significant effect of group (F = 74.687, p < 0.001). Post hoc tests (Holm–Sidak method) showed that the between-group differences were significant at every frequency tested (t = 2.08, P = 0.04 for 2k Hz; t = 3.75, P < 0.001 for 4k Hz; t = 3.13, P = 0.002 for 8k Hz; t = 8.44, P < 0.001 for 16k Hz; and t = 2.86, P = 0.005 for 32k Hz).

**Figure 2:**
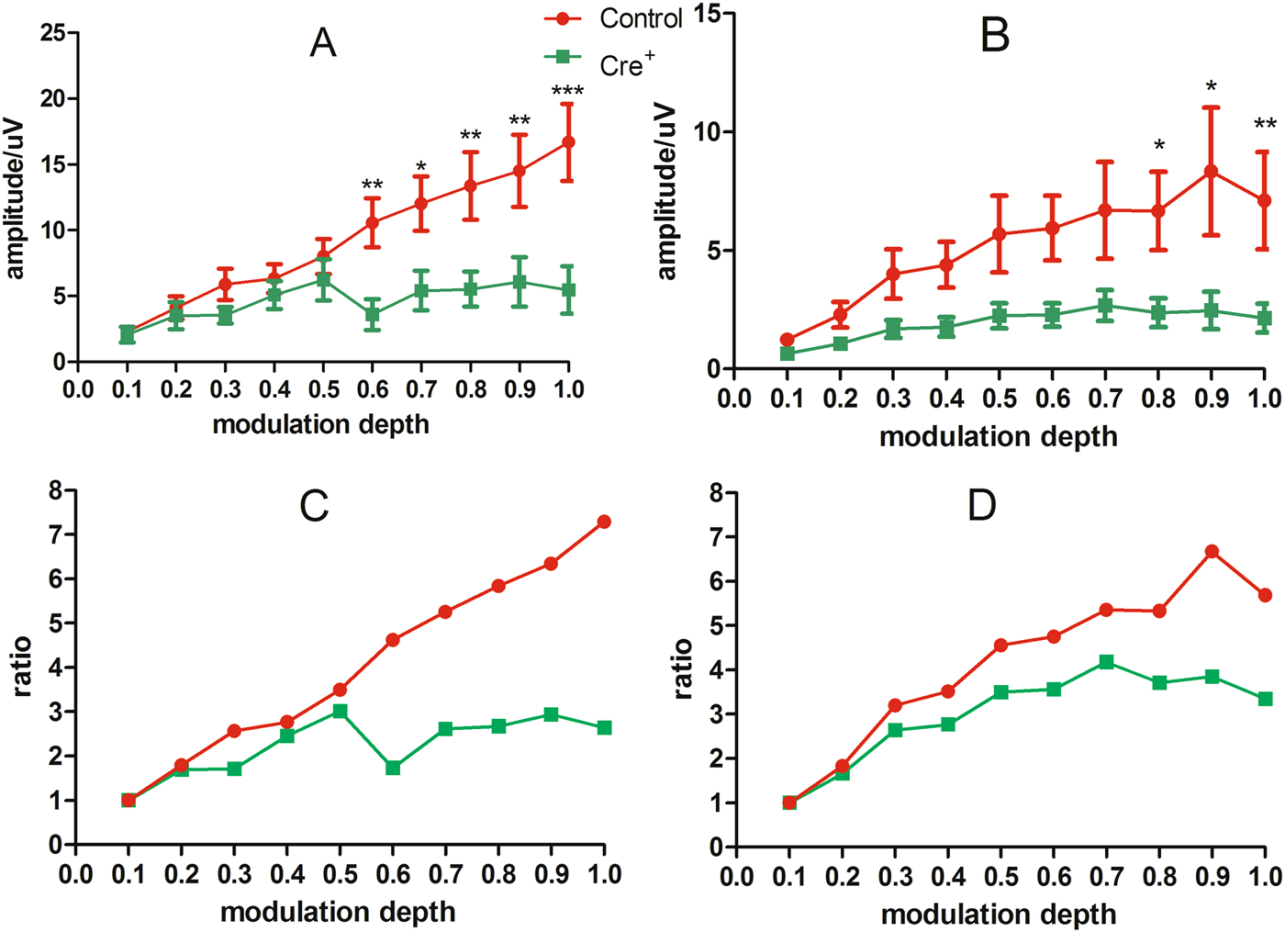
The impact of DT-induced damage on AM response. The frequency of the carrier was 16 kHz. The AM CAP were measured with Fm of 112 Hz (**A**,**C**) and 996 Hz (**B**,**D**) respectively, and shown as the amplitude (**A** and **B**) and ratio change (**C** and **D**) as the function of modulation depths in percentage. The AM CAP was significantly smaller in Cre^+^ group, especially at larger modulation depth. *p < 0.05, **p < 0.01, and ***p < 0.001.

To evaluate ANF synchronization to auditory stimulation, we recorded CAPs evoked by AM and compared between the *Cre* ^*+*^ and control group (n = 10 in each group). The carrier frequency (Fc) was 16 kHz, and the modulation frequency (Fm) was 112 and 996 Hz, respectively. At each Fm, the response amplitude was tested as a function of modulation depth from 10% to 100% in 10% steps. The overall impression is that, while the response amplitude was largely increased with greater modulation depth in the control group, the corresponding change in the *Cre* ^*+*^ group was very small and became saturated at a modulation depth above 50% (Fig. 2A and B). This saturation is further demonstrated in the normalized ratio–modulation depth functions (Fig. 2C and D). In the control group, the AM response amplitude was increased from 2.29 ± 0.34 μV at 10% modulation to 16.69 ± 2.93 μV at 100% modulation for the modulation frequency of 112 Hz, representing a change of 7.29 fold (Fig. 2C); the change was 5.68 fold for Fm at 996 Hz (Fig. 2D). However, the corresponding changes in the *Cre* ^*+*^ groups were only 2.64 and 3.34 fold, respectively, for the two modulation frequencies. A two-way ANOVA was performed using genotype as the main factor and depth as a covariate. The results showed that there was a significant main effect of genotype on AM amplitude (for modulation frequency = 112 and 996 Hz, F = 33.601, *p* < 0.001 and F = 27.531, *p* < 0.001, respectively). Post hoc tests (Holm–Sidak method) were performed between groups at each modulation depth and the significance level is denoted by asterisks in Fig. 2.

### Cochlear morphology

**Figure 3:**
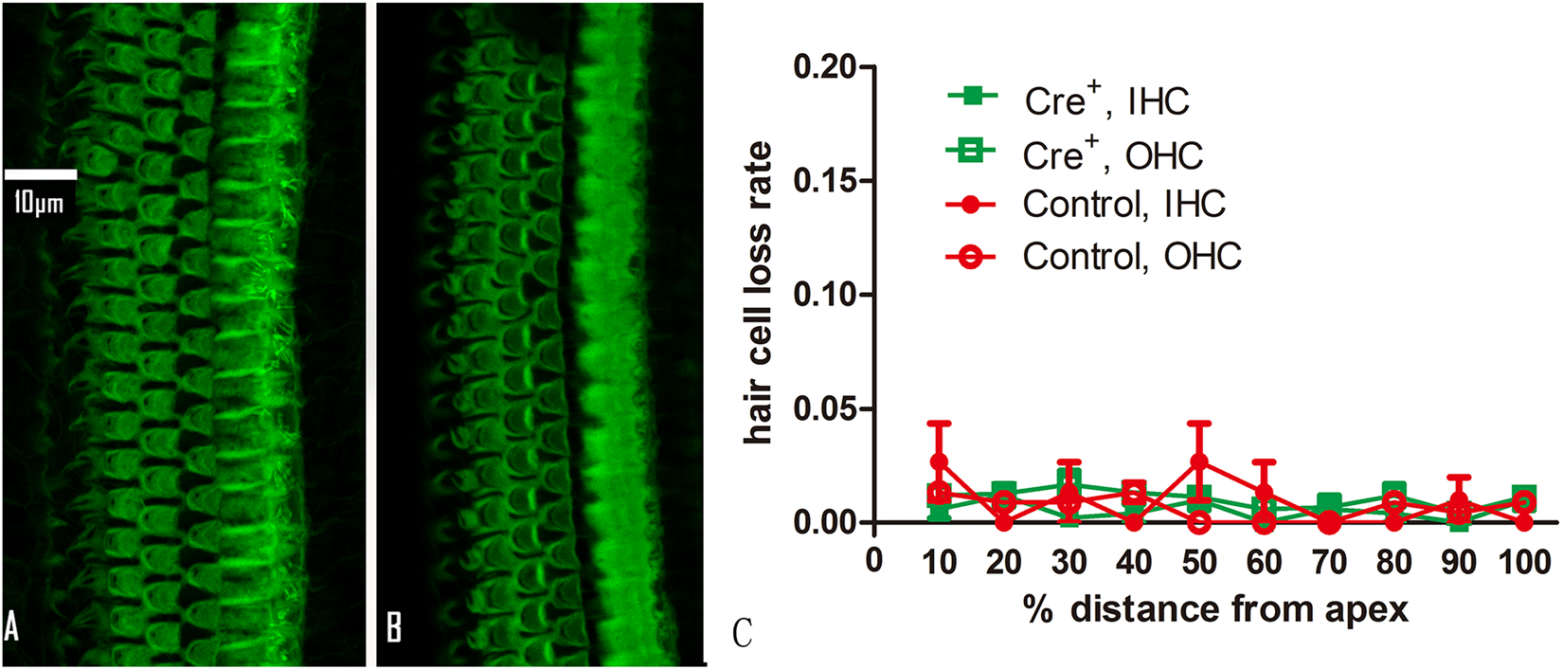
The DT impact on HCs. (**A** and **B**) Representative images of HCs in the surface preparation of the basilar membrane from the control and the Cre^+^ group respectively. HCs are stained green with antibody against actin. (**C**) The averaged cochleograms of both groups showing percentage HC loss as a function of percentage distance from the apex.

Corresponding to the no ABR threshold changes in *Cre* ^*+*^ animals, we found no significant hair cell loss in cochleograms, which were generated from samples stained with phalloidin–rhodamine (Fig. 3).

**Figure 4:**
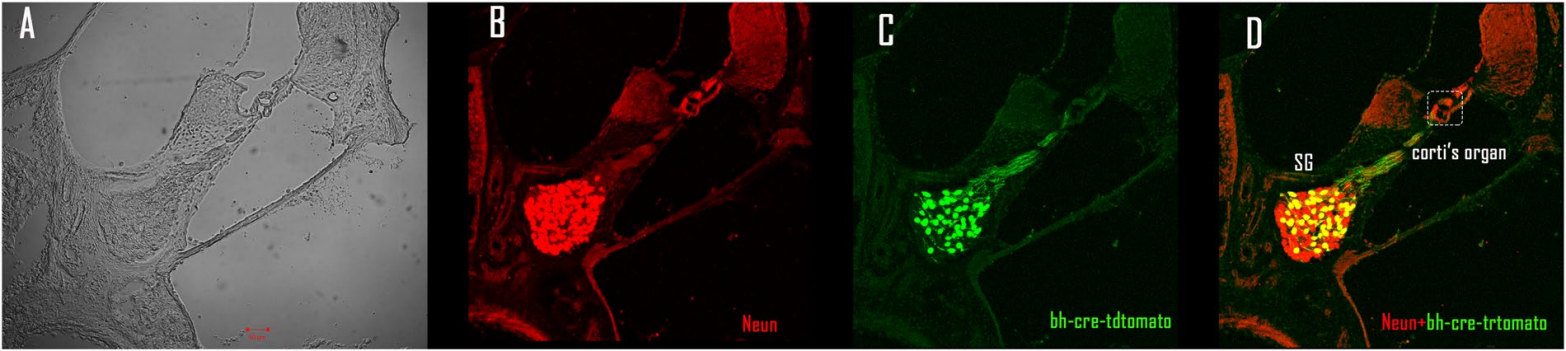
Images identifying SGN specific expression of Bhlhb5-cre. A cochlear cross section slide was observed (unstained) under the light microscope (**A**), and then stained with Neun (**B**), and against tdTomato (**C**) to show the Cre. (**D**) Merged images of (**B** and **C**).

To confirm the location of Cre expression, we used a mouse line with Cre reporter protein tdTomato (*bhlhb5-cre-tdTomato*)^19^. Green florescence of tdTomato (Fig. 4C) was observed only in cells labeled with the neuron marker NeuN, suggesting that Cre was selectively expressed in SGNs.

**Figure 5:**
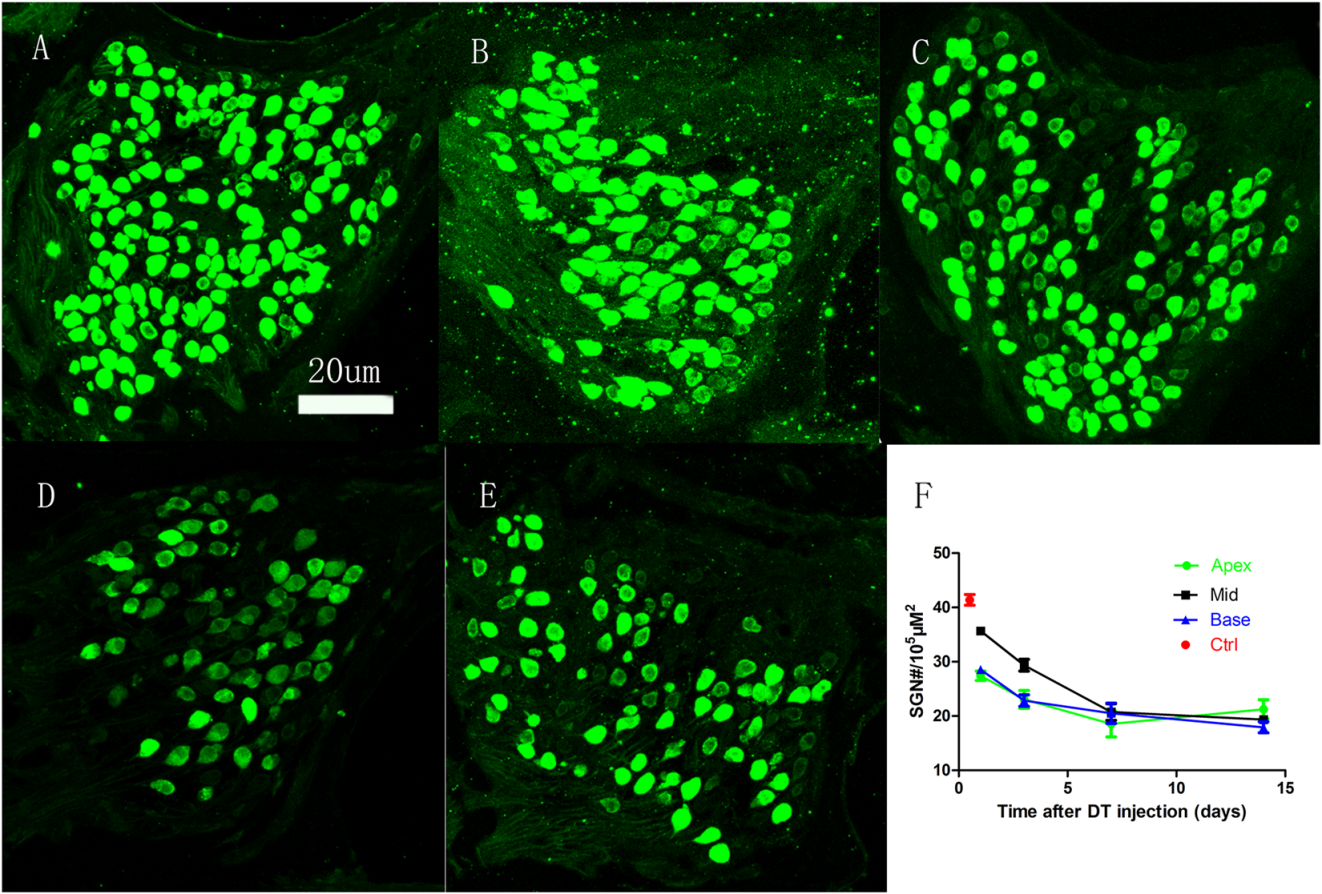
DT-induced changes in SGN density. Representative images of SGNs in Rosenthal’s canal at the basal turn acquired from control subjects and Cre^+^ subjects measured at 1, 3, 7, and 14 days after the DT injection (**A**–**E**, respectively). Quantitative changes of SGN density at the three locations (apex, middle, and basal) as a function of time after the DT injection (**F**).

The dynamic loss of SGNs caused by DT injections in the *Cre* ^*+*^ group is illustrated in Fig. 5. Figure 5A shows a representative image of the basal Rosenthal’s canal from the control group, and Fig. 5B–E show representative images obtained from the *Cre* ^*+*^ group at the same location at different time points after DT injection (1, 3, 7, and 14 days, respectively). Figure 5F shows changes in SGN density at three locations in the cochlea (apex, middle, and base) of the *Cre* ^*+*^ group according to time after DT injection. We summarized the change of SGN density in Table 1. Although the SGNs were tightly packed in Rosenthal’s canal in the control group, they were much less closely packed in the *Cre* ^*+*^ samples (Fig. 5B–E). The number of SGNs in each view was determined and then divided by the area of Rosenthal’s canal to calculate SGN density (#SGNs/um^2^); in Fig. 5F, the control SGN density was pooled across the three locations because there were no significant differences among the values. A rapid decrease in SGN density was observed at approximately 3 days after the DT injection; however, the change between 3 and 7 days was small, and there was almost no change between 7 and 14 days. A two-way ANOVA performed using time after DT injection as the main factor (0 days as the control and 1, 3, 7, and 14 days to represent a combination of the group and time delay after the DT injection) and location as the covariate. The effect of the main factor was significant (F = 103.88, *p* < 0.001), but the effect of location was not. Post hoc tests (Holm–Sidak method) revealed that the SGN densities measured at each of the four time points and three locations were significantly lower than the control values (p < 0.001). For each location, the densities measured at 3 and 7 days were significantly lower than those measured at the previous time point, but there was no significant difference between 7 and 14 days at any of the locations.

**Table 1:** Between-group comparison on SGN density.

group	control	1 dpi	3 dpi	7 dpi	14 dpi
location					
apical	41.39 ± 1.00	27.40 ± 0.87	23.08 ± 1.65	18.54 ± 2.38	21.24 ± 1.79
middle	40.93 ± 1.57	35.69 ± 0.56	29.35 ± 1.09	20.76 ± 1.53	19.35 ± 0.70
basal	40.93 ± 1.47	28.54 ± 2.17	22.84 ± 1.07	20.51 ± 1.87	17.92 ± 1.02

N > 5 for each group. The SGN density is measured as the SGN#/10000 nm^3^. The density measured in Cre^+^ group at every time point after DT injection was significantly lower than the control value at each location (p < 0.001).

**Figure 6:**
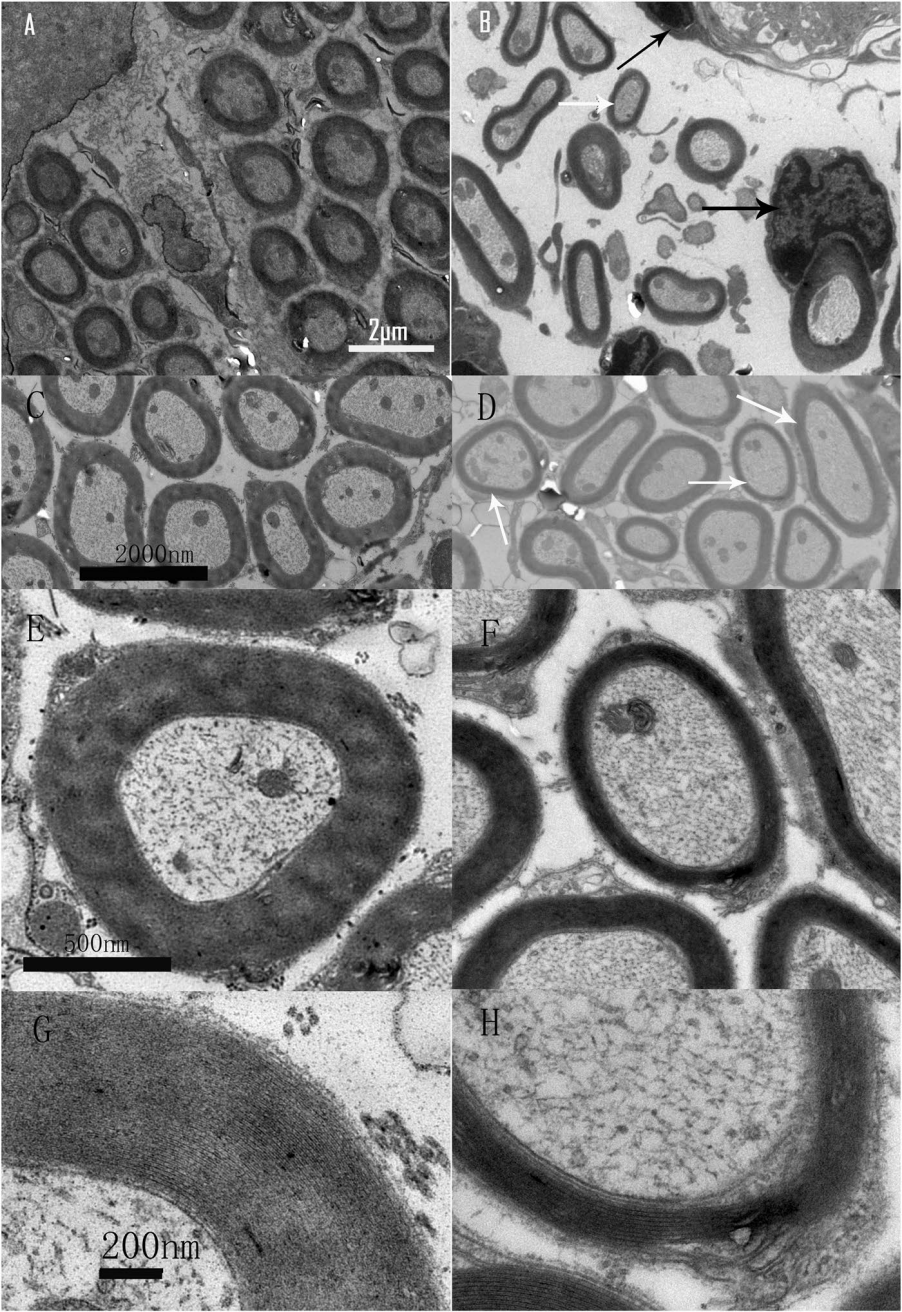
TEM images showing DT-induced ANF damage. Magnification was 6000×, 20000×, and 50000× in A–D, E and F, and G and H, respectively. The images in the left and right columns are from the control and Cre^+^ groups, respectively. The cross sections of ANFs were in regular round shape (**A**,**C**) with relatively even thickness of myelin sheaths, in contrast to the irregular shape and uneven thickness of myelin sheaths in the images from the Cre^+^ group (**B**,**D**). The white arrows in (**B** and **D)** point to nerve fibers whose myelin sheath was peeling off. Additionally, extremely swollen Schwann cells were seen in the Cre^+^ sample (black arrows in **B** and **D**). The images of higher magnification (**E**–**H**) show that the myelin sheaths in the Cre^+^ sample is thin (**F**) and loosely packed (**H**) as compared with the control sample (**E** and **G**).

Morphological abnormalities in the SGNs were assessed using transmission electron microscope (TEM). In addition to the debrides of broken nerve fibers, one striking piece of evidence that DT damaged SGNs in the *Cre* ^*+*^ group was swollen and darker Schwann cells (Fig. 6B). Whereas the cross-section views of the SGN fibers in the control samples were mostly rounded and were surrounded by tightly packed myelin sheaths of even thickness (Fig. 6A and C), the fibers in the *Cre* ^+^ group showed significant variation in shape and in the thickness of the myelin sheath. The thickness of myelin sheath was 211.93 ± 8.24 nm in the *Cre*+ group, which was significantly thinner that the value of 345.94 ± 11.50 nm in the control groups (t = −9.23, p < 0.001). Moreover, disruptions of the myelin sheath were frequently seen (white arrows in Fig. 6B and D and Supplementary Fig. S1).

## Discussion

The major findings of the present study were as follows: 1. Roughly 40% of SGN loss at 7 days post DT injection in adult mice in which DTR was induced in SGNs was due to specific Cre; the SGN loss did not increase thereafter; 2. Hair cells were not damaged in this model and the auditory sensitivity remained unchanged; 3. The amplitude of evoked potentials from ANFs were significantly decreased, by 50% – which is slightly higher than that of SGN loss – suggestive of a functional abnormality of surviving ANFs; 4. Most importantly, a significant reduction in ANF responses to AM was observed in the SGN-damaged *Cre* ^*+*^ group, suggestive of a significant loss in response synchrony to the temporal envelope of sound; and 5. Correspondingly, demyelination-like pathologies were observed in the *Cre* ^*+*^ group in addition to the breakdown of ANFs following SGN death.

We believe that our model can be used as an animal model for AN with demyelination because we show a clear loss of synchronous response of auditory nerves, to the temporal envelope of sound, in association with selective damage to SGNs and their fibers. Although no clear morphological evidence of ANF demyelination is available, ANF dyssynchrony appears to be the major problem and considered to be linked to ANF demyelination in human AN subjects^8, 20, 21^. The development of demyelination model as such in the present study will help to verify if and how much ANF demyelination contributes to AN-like problems in signal processing.

Since the evoked responses from the lower level stations in the ascending auditory pathway are generated from a relatively smaller number of neurons located deep inside the brain, and are distant from the skull surface electrodes, responses seen in far-field recordings more heavily rely on synchrony of the responses as compared to their central counterparts. Therefore, responses such as ABR can only be induced by transient stimuli. Responses to AM signals are useful to show the ability of auditory neurons to produce synchronous responses to the temporal envelope of the acoustic signals^1, 13^.

Dyssynchrony may occur as a result of (1) synaptopathy involving pre- and/or postsynaptic parts of ribbon synapses between IHCs and SGNs, and/or (2) demyelination of the auditory nerve. In our model of DT-induced damage with SGN-specific Cre, the major pathology of surviving ANFs was demyelination. This was demonstrated by the uneven thickness of the myelin sheath and the distorted shape of nerve fibers in the Cre^+^ group (Fig. 6). More examples of damage to ANFs are provided in the supplementary figures (Supplementary Fig. S1).

It remains unclear whether the SGN damage in our model was biased to the low-SR units, as seen in recent studies on noise-induced hidden hearing loss (NIHHL)^22^. Such a bias was observed in previous studies using ouabain, in which the damage to ANFs did not impact the threshold or CAP amplitude if ouabain was applied at a low dose^23^. It is suggested that, at this low dose, only ANFs with very high thresholds were damaged so that the threshold was not impacted. Since the CAP amplitude was not changed at this dose, the authors concluded that the damaged ANFs do not contribute to the onset response. A limited increase of ouabain would kill more ANFs and decrease the amplitude but still not change the threshold. In our model, further investigations are required to determine whether there is any synaptopathy and if the damage to ANFs is selective or biased to the low-SR units as seen in the ouabain model.

Efforts have been made to identify an effective and reliable method to detect functional loss of low-SR ANFs in NIHHL^1, 24^. Transient auditory responses such as ABR and CAP rely on the synchrony of ANFs to the signal onset. It is believed that the low-SR ANF units are less important or do not significantly contribute to the onset responses^1, 25^. However, there is a lack of information on whether and why the low-SR ANFs were less effective at coding the onset of sound and did not contribute to CAP, which is the transient response to onset. In one of our recent studies on NIHHL in guinea pigs, we did observe a significant difference in the onset responses of ANFs of different SRs^3^. On the other hand, AM responses tested at relatively high sound levels are likely to be more sensitive in detecting neural dyssynchrony. Different strategies have been proposed for this purpose^1, 24^. In a study using far field recording, dyssynchronous malfunction was reported to be better detected using Fm close to 1 kHz^1^. In the present study, we used Fm = 112 and 996 Hz to evaluate the synchrony of CAP to AM with a carrier at 16 kHz. In addition, the differences between groups were observed at relatively larger modulation depths. This conflicted with what was recently predicted by others^24^. Moreover, our results did not show an advantage of using 996 Hz Fm as seen in the far field recording in the mouse model with NIHHL. Since the AM responses at the supra-threshold levels were poorer in the Cre^+^ group, it is likely that the SGN damage is also biased toward those units with low SR and larger dynamic ranges.

The selective damage to SGN was confirmed based on cochlear images from the *Bhhlb5-cre-tdTomato* mice (Fig. 4). In this study, we used tdTomato to report the expression of Cre. The immunohistological identification confirmed that the Cre expression was selective to SGNs. Since DTR was expressed only in SGNs (not in Schwann cells), we believe that the damage to Schwann cells and demyelination were secondary to SGN damage. This is supported by the fact that the intact morphology and functions of glia cells depends on the health of the neurons they cover^26, 27^. Since the surviving nerve fibers showed irregular shape and reduced diameters in the *Cre* ^*+*^ group (Fig. 6B,D and F), it is believed that they were damaged.

In this study, we did not test OAE, which is an objective indicator of OHC electromotility. Since OHCs contribute mainly to and are required for normal hearing thresholds^28^, OAE is likely to be normal in the Cre^+^ group when the thresholds remain unchanged.

The present study had several limitations that need to be addressed in the future. First, the demyelination seen in this model is likely secondary to the SGN and nerve fiber damage. Second, the potential damage to synapse function was not verified. Third, our observations were performed at only one time point, so the long-term fate of the SGNs that survived remains unclear.

In conclusion, our study demonstrates that mice with iDTR conditionally expressed in SGNs by bhhlb5-Cre represent a good model for AN, and this model can be used to study the molecular mechanisms underlying AN in the future.

## Materials and Methods

### Subjects and experimental outline

The mouse lines with *iDTR* (*Rosa26iDTR*)^29^ and tdTomato (*ROSA26* ^*CAG-tdTomato/+*^) were obtained from the Jackson Laboratory (Bar Harbor, ME, USA) (Cat# 007900 and Cat# 007908). The mouse line with SGN-specific Cre (*Bhlhb5-Cre*)^30^ was a gift from Dr. Lin Gan (Flaum Eye Institute and Department of Ophthalmology, University of Rochester, Rochester, NY, USA). The method of genotyping *ROSA26* ^*CAG-tdTomato/*+^, *Rosa26iDTR*, and *Bhlhb5-Cre* was described previously^19, 29, 30^. To induce selective and conditional SGN death in the cochlea of adult mice, we cross-mated the mouse line with SGN-specific Cre (*Bhlhb5-Cre* ^+/−^) to the mouse line with Cre-inducible diphtheria toxin receptor (*iDTR* ^+/+^). The first generation of offspring were used as the subjects in this study, in which all mice had a genotype of *iDTR* ^+/−^. Those with no *Bhlhb5-Cre* (*Bhlhb5-Cre* ^*−*^) were used as controls and those that were *Bhlhb5-Cre* ^*+*^ were used as experimental subjects in which Cre-mediated excision of the floxed stops sequence in the *ROSA26-loxP-stop-loxP-DTR* (*ROSA26DTR*) allele causes selective DTR expression in *Cre* ^+^ cells (SGNs in cochleae). The specificity of such Cre to SGN was confirmed using *Bhlhb5-Cre-tdTomato* mice (Fig. 4).

In total, 40 mice (both genders) were used in this experiment with 20 each assigned to the control (Cre^−^) and experimental (Cre^+^) groups. All subjects in both groups received one intraperitoneal injection (i.p.) of DT at a dose of 25 ng/g (List Biological Laboratories, Campbell, CA, USA) at postnatal day 21. Seven days after the DT injection, all functional evaluations were performed, including ABR, CAPs to both tone bursts, and AM signals. The functional tests were followed by a count of HCs and observation of SGN morphology using both LM and TEM. All animal procedures were approved by the University Committee of Laboratory Animals of Shanghai Jiao Tong University, China (Permit Number: SYXK (H2011-0128)). This protocol was developed in accordance with the National Institutes of Health Guidelines for the Care and Use of Laboratory Animals (NIH publications N. 8023).

### Functional tests

Prior to the ABR and CAP recordings, the animals were anesthetized using ketamine mixed with Xylazine (80 mg/kg and 10 mg/kg, i.p.). During the recording, the body temperature of the animals was maintained at 38 °C with a thermal static heating device (FHC; MA, USA). Three subdermal needle electrodes were used to record the ABRs: the non-inverting electrode was inserted at the vertex, and the reference and grounding electrodes were inserted at the neck posterior to the auditory bullas of both sides. To record the CAPs, a silver ball electrode was placed at the round window membrane via a small hole drilled on the auditory bulla posterior-inferior to the external ear canal. The other end of the electrode was led to the non-inverting channel of the pre-amplifier.

Hardware and software from Tucker-Davis Technologies (TDT System III; Alachua, FL, USA) were used for stimulus generation and bio-signal acquisition. The acoustic stimuli used were as follows: (1) 10-ms tone bursts for ABR and CAP with cos^2^ gating and a 0.5-ms rise/fall time, and (2) AM tones of 500 ms duration (rise/fall time 5 ms) for AM CAP. The stimuli were played through a broadband speaker (MF1; TDT) that was placed 10 cm in front of the heads of the animals. The evoked responses were amplified 20 fold and then filtered between 100 and 3,000 Hz using a preamplifier (RA16PA; TDT), which digitized the signal at a sampling rate of 25 kHz. The responses were averaged 1,000 times for ABR and 100 times for CAP. The ABR thresholds and the tone-burst CAP input/output (I/O) functions were tested at 2, 4, 8, 16 and 32 kHz. The stimulation rate for tone burst-evoked ABR and CAP was 21.1/s. At each frequency, the test was started at 90 dB SPL and tracked in 5-dB steps until the response disappeared. In the ABR test, the focus was the thresholds, which were defined as the lowest level at which a repeatable wave III response could be obtained. In the CAP tested with tone burst, the focus was the peak-to-peak amplitude, which was measured at different sound levels to generate I/O functions. The CAP response to AM was recorded with sweeping presentation of an AM tone at a rate of 1.2/s. The first 50 ms of the response in the 500 ms time window was set to zero to eliminate the effect of the onset response. The remaining responses were averaged and converted into the frequency domain spectrum with a resolution of 1.49 Hz. The signal amplitude was tested as the peak amplitude in the spectrum at Fm.

### HC morphology

Following the endpoint functional tests, the animals were sacrificed and the cochleae were harvested when the animals were deeply anesthetized with pentobarbital (100 mg/kg, intramuscular). In total, 10 cochleae (one from each animal) in each group were used in the HC count. To generate the HC cochleogram, the cochleae were quickly harvested and perfused rapidly three times with 4% paraformaldehyde in phosphate-buffered saline (PBS). They then underwent a brief 1 h post-fixation at 4 °C followed by decalcification in 10% EDTA at room temperature for 6–10 h. Next, each cochlea was transferred to PBS, and the bone shell of the cochlea was removed using fine forceps. The cochlea was then treated with phalloidin–rhodamine (1:400) P1951; Sigma, St. Louis, MO, USA) for 2 h at room temperature. Following the staining procedure, the basilar membrane of each cochlea was dissected into three pieces, mounted onto microscope slides, and coverslipped. Confocal images were acquired using a confocal laser-scanning microscope (LSM 710 META; Zeiss, Shanghai, China) with 100× oil-immersion objectives. Image stacks were then exported to image-processing software (Lsmix and ImageJ).

### Test for Cre expression

We used the mouse line with SGN-specific Cre (*Bhlhb5-Cre* ^+/−^) to cross mate with a mouse line having a Cre reporter locus *ROSA26* ^*CAG-tdTomato*^. The first offspring mice are *Bhlhb5-Cre* ^+, *tdTomato*^, in which the expression of Cre recombinase will turn on the reporter protein, tdTomato, with a strong fluorescent signal^19^. One such animal was sacrificed by decapitation and both cochleae were dissected and perfused with a fixative solution containing 4% paraformaldehyde in PBS (pH 7.2–7.4) for 1 h at 4 °C. The apical portion of the bony cochlea was gently opened to allow the fixative to be perfused through the cochlea. After rinsing (2 × 5 min) in PBS, the cochleae were decalcified overnight in EDTA solution at room temperature and then soaked in 15% and 30% sucrose solution for 8 h. The cochleae were placed in Tissue-Tek OCT compound (Tissue-Tek, Labonord, France) for cryostat sections. The cochleae were oriented to obtain cross-sections of the Rosenthal canal. The sections were cut to 14 μm thickness by cryostat and mounted on Premiere^®^ slides. Sections were then permeabilized with 0.01% Triton X-100 in PBS for 30 min, incubated for 30 min in 5% goat serum in PBS, incubated in primary antibody (1:200) (rabbit anti-NeuN, cat#12943; CST; MA, USA) overnight at 4 °C, and then incubated in secondary antibodies (1:800) (goat anti-rabbit, A11034; Invitrogen, Shanghai, China) for 2 h at room temperature. All antibodies were diluted in 5% goat serum in PBS. Confocal images were acquired using a confocal laser-scanning microscope (710 META; Zeiss) with 10× objectives and the image stacks were ported to image-processing software (Lsmix and ImageJ).

### SGN morphology

The SGN count was performed using a total of 25 animals from the control group and the *Cre* ^*+*^ group at different time points following the DT injection.

In each animal, one cochlea was used to obtain a frozen section for immunofluorescence observations; the method was introduced earlier in the present paper (Test for Cre expression). The number of SGNs was determined using confocal microscopy at three different locations across the basilar membrane (15%, 40%, and 75% from the apex for the apical, middle, and basal parts of the cochlea, respectively). At each of these three locations, the SGNs were counted in four slices (each containing a single view of Rosenthal’s canal) over a distance of 56 μm. In each view, the area of Rosenthal’s canal was calculated using ImageJ, and the number of SGNs was counted and converted to an SGN density value (#SGN/um^2^).

To observe ultra-structural changes in the SGNs, the cochleae of the control group and of each experimental animal at 7 days after the DT injection were used for ultra-thin dissection for TEM. After functional testing, the animal was sacrificed under deep anesthesia and the cochleae were harvested. The cochlea was perfused with 2% glutaraldehyde in PB buffer and then immersed in the fixative for 6 hours at 4 °C, followed by decalcification in 10% EDTA for 7 days at 4 °C. After further fixation in 1% osmium acid for 2 hours at room temperature and dehydration in grade ethanol, the sample was embedded with Epon using standard procedures. Next, semi-thin cross sections (1 μm) were made to confirm the accuracy of the locations.

Ultra-thin (70 nm) sections of the Rosenthal canal, around the 16 kHz region on the cochlear frequency map, were prepared^31^. The canals were cut in parallel with the long axis. Once the cochlea spiral ganglion cells were observed, several ultrathin silver-to-gray sections were collected on Formvar films on single-hole copper grids. The sections were then stained with 2% aqueous uranyl acetate (19481; Ted Pella, Inc., Redding, CA, USA) and lead citrate (19312; Ted Pella). The samples were observed and images were taken with a TEM (80 kV) (Tecnai Spirit; FEI, Hillsboro, OR, USA) at 6,000×, 20,000×, and 50000× magnification to observe the entire SGN and the myelin sheath. We took the TEM micrograph which represented the transverse section of ANFs. Then we measured the thickness of myelin sheath by ImageJ software on such TEM micrographs. Data were evaluated form more than 60 myelinated axons from each group for assessment of myelin thickness (N = 3 animals for each group).

### Statistical analysis

All data are expressed as means ± standard error of the mean (SEM). Grouped t-test and two way-ANOVA were performed using SAS software (SAS Institute Inc., Cary, NC, USA). A *p* value < 0.05 was taken to indicate a significant difference in all tests.

## Electronic supplementary material

Supplementary Information
