# Supplementary material for: Auditory Neuropathy after Damage to Cochlear Spiral Ganglion Neurons in Mice Resulting from Conditional Expression of Diphtheria Toxin Receptors
Source: Sci Rep. 2017 Jul 25;7:6409. doi: 10.1038/s41598-017-06600-6 (PMC5527113; doi:10.1038/s41598-017-06600-6)
Supplement: Supplementary file 1 — Supplementary Information [file 41598_2017_6600_MOESM1_ESM.pdf]

Auditory Neuropathy after Damage to Cochlear Spiral Ganglion Neurons in Mice Resulting from Conditional Expression of Diphtheria Toxin Receptors

Haolai Pan1, Qiang Song1, Yanyan Huang1, Jiping Wang1, Renjie Chai2, Shankai Yin1*, Jian Wang1,3*

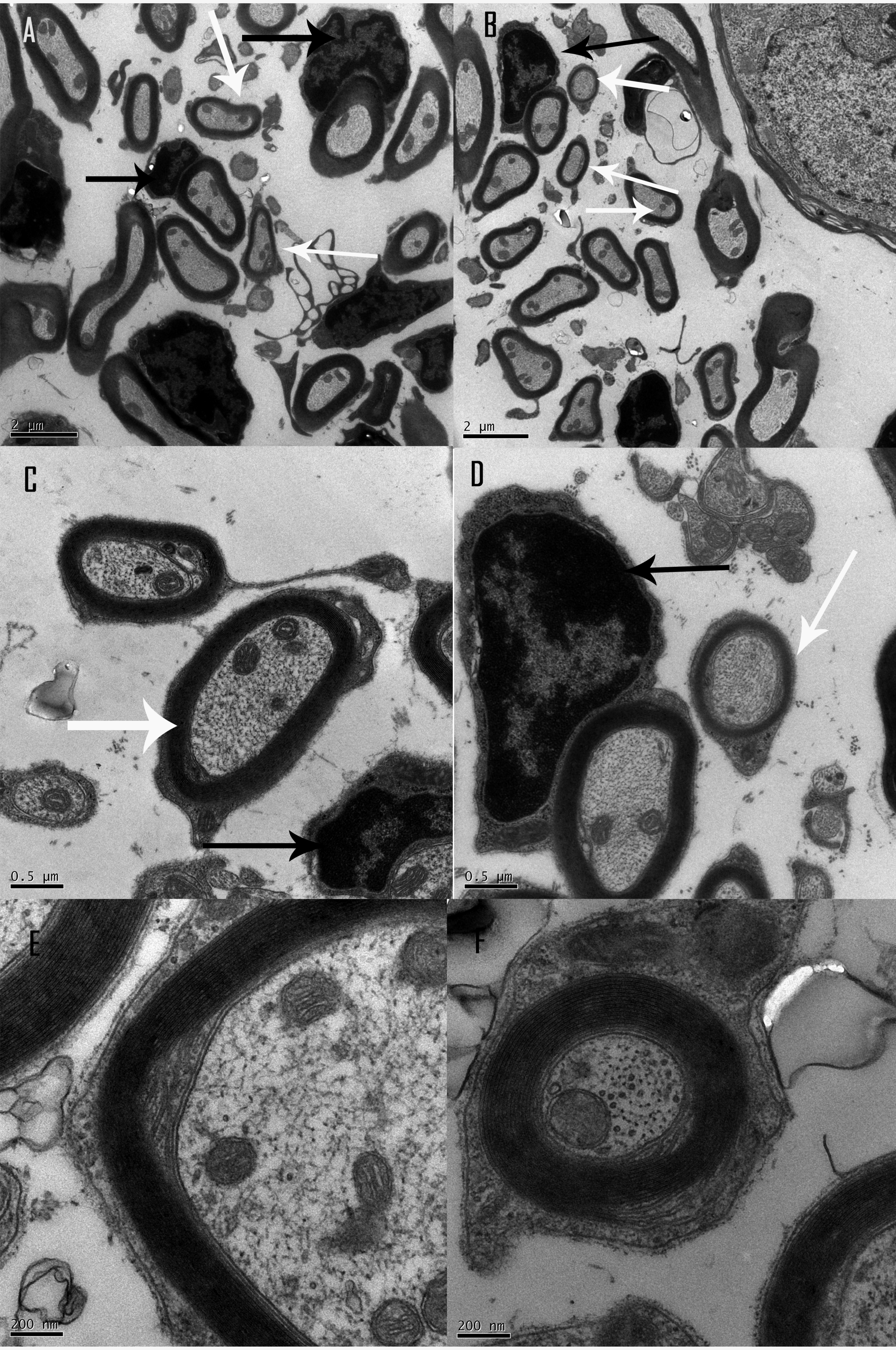

sFigure S1. More TEM images of ANF from samples of the Cre+ group.

Black arrows pointed to Schwann cells that were extremely swollen, probably were in the process of apoptosis of Schwann cells. White arrows pointed to the myelin sheath with different thickness. The cross-section of fibers showed different shapes.
